# Interpersonal reactivity: the impact of infant-juvenile positive experiences

**DOI:** 10.1080/07853890.2021.1896177

**Published:** 2021-09-28

**Authors:** Carolina Duarte, Rita Fatela, Telma C. Almeida

**Affiliations:** a Instituto Universitário Egas Moniz (IUEM), Egas Moniz Cooperativa de Ensino Superior, Caparica, Portugal; b Laboratório de Psicologia Egas Moniz (LabPSI), Centro de Investigação Interdisciplinar Egas Moniz (CiiEM), Egas Moniz Cooperativa de Ensino Superior, Caparica, Portugal

## Abstract

**Introduction:**

Children's exposure to negative and positive experiences has consequences throughout their lifetime [1]. Positive experiences are associated with how the child sees and interacts with their world [2], and it helps to develop mitigating factors for the adverse effects of adverse experiences and promoters of resilience [3]. The objectives of this research are to study the relationship between interpersonal reactivity (empathy) and positive experiences in childhood and to compare victims and non-victims of traumatic events.

**Materials and methods:**

The study design is descriptive, observational, and cross-sectional. The study was conducted in accordance with the ethical principles outlined in the Declaration of Helsinki [4]. Participants responded to the protocol in the google form, having consented to their participation in order to advance in the protocol. This study comprised 147 Portuguese adults aged between 18 and 67 (*M* = 30.8, *SD* = 11.6). The link to the study was disclosed by e-mail and in social networks. Samples were divided into two groups: G1 – non-victims of traumatic events (*n* = 84) and G2 – victims of trauma in the last three years (*n* = 63). Participants responded online to a sociodemographic questionnaire, the Interpersonal Reactivity Index (IRI) [5], and the Benevolent Childhood Experiences (BCEs) scale [6].

**Results:**

Data showed statistically significant correlations between: Interpersonal Support and the Perspective Taking (*V* = 0.402, *p* = .035); Interpersonal Support and Empathic Concern (*V* = 0.371, *p* = .038); the Perceived Relational and Internal Safety and Security, and Fantasy (*V* = 0.438, *p* = .035); the total score of the BCEs and the subscale Fantasy in the IRI (*V* = 0.421, *p* = .036). Concerning the experience of traumatic events in adulthood, the results revealed statistically significant differences. Compared to G2, the G1, highlighted higher levels of total score of the BCEs [*F*= (1,145) = 5.07, *p* = .026]), Positive Experiences of Support [*F* = (1,145) = 6.02, *p* = .015]), and Positive Experiences of Security [*F* = (1.145) = 4.30, *p* = .040]) in infant-juvenile stage.

**Discussion and conclusions:**

This research points to an association between the existence of positive experiences in the infant-juvenile phase and the development of empathy in adulthood. Some studies corroborate our results, demonstrating that the experiences in the first stages of life will have long-term repercussions on their social-emotional development [3]. Since empathy and positive experiences interfere in child development, that's important to promote, reinforce and safeguard these experiences throughout the stages of the life cycle.
